# 

**Published:** 1882-09

**Authors:** 


### TO BE RESUMED

### THE MAD RIVER DENTAL SOCIETY.

This society, the meetings of which have been suspended for several years, is to be revived. It has not been dead, but only sleeping, as I suspect, to gather strength. This was one of the few societies that was not annoyed by ax-grinding; at least, till about its last meeting when somebody brought in a broad-ax and ground at it nearly all one session, and it so discouraged the society that it went to bed sick and has been there ever since. It is now thought to be convalescent and able to resume business. And so on the occasion of a Fourth-of-July visit by some of the old members to Dr. Geo. Watt, at Xenia, it was decided to put the little society on its feet again, and so the following programme has been agreed upon:

The first meeting will be held in Dayton, O., on Tuesday, October 24, at 10 o'clock A. M., till 12 M. Second session, from 2 till 5½ P. M. Evening session, from 7 till 10 P. M.

At 10 o'clock A. M., organization and election of new members, and following this election of officers.

1st. A historical sketch of the society, will probably be presented by Dr. Geo. Watt.

2d. Dental colleges, by Dr. A. Berry.

3d. Replanting teeth, by Dr. Will Taft.

4th. Prosthetic dentistry, by Dr. C. M. Wright.

It is to be hoped that all the members of the profession within fifty miles of Dayton will devote the day named to the Mad River Society, and let every one bring something that will interest every one else.

### THE DENTAL REGISTER,

A MONTHLY MAGAZINE DEVOTED TO THE INTERESTS OF THE

**DENTAL PROFESSION.**

*Terms Reduced to $2.00 Per Year. Single Copies, 25 Cents.*

**EDITED BY PROF. J. TAFT, D.D.S.**

AND

***Published by SPENCER & CROCKER, Ohio Dental Depot.***

The volume commences in January and closes in December.

The REGISTER being issued monthly is always fresh, therefore Indispensable to the practitioner who desires to keep posted up with the new inventions and improvements which are daily developed. Neither expense nor effort will be spared to give value and variety to its contents. Articles will be illustrated with well executed engravings whenever they will add to the interest or assist to explanation.

Its extensive circulation and frequency of issue make it one of the BEST DENTAL ADVERTISING MEDIUMS in the United States.

All subscriptions must begin with January or July.

Local or special notices, five lines or less, will be inserted for $3 each insertion; over five lines, 50 cts. per line.

All advertising bills payable in advance, or at furthest, after the issue of the first number containing the advertisement. All transient advertisements to be paid for in advance.

☞CONTRIBUTIONS SOLICITED.

Exclusively Editorial Communications should be addressed to the Editor.

The latest number of the REGISTER may be ordered with or without other goods at any time, and all letters should be addressed to

**SPENCER & CROCKER, Ohio Dental Depot, Cincinnati, Ohio.**

